# Editorial: Modern treatment of ventricular arrhythmias

**DOI:** 10.3389/fcvm.2022.1109993

**Published:** 2023-01-04

**Authors:** Simone Savastano, Roberto Rordorf

**Affiliations:** Division of Cardiology, Arrhythmia and Electrophysiology, Fondazione Istituto di Ricovero e Cura a Carattere Scientifico (IRCCS) Policlinico San Matteo, Pavia, Italy

**Keywords:** ventricular arrhythmias, catheter ablation, electric storm, stereotactic ablation, neuromodulation

Ventricular arrhythmias (VA) encompass a wide spectrum of clinical conditions, ranging from benign conditions, such as isolated ectopic beats in patients without structural heart disease, to life-threatening emergencies, such as electrical storms in patients with very advanced structural heart disease. Owing to the great variety of clinical conditions, pathophysiological mechanisms, and therapeutic approaches, VA have been the focus of a thriving body of literature.

A bibliometric analysis by Wang S. et al., presented in this special issue, shows that in the last 20 years, ~7,000 papers have been published about VA, with “catheter ablation” being one of the main topics. A large amount of research contributed to the latest update of European guidelines on VA and sudden death (1), where catheter ablation earned quite a high number of class I and IIa indications, thanks to technological advances driving important results of clinical trials.

Catheter ablation may also be curative in clinical situations that are challenging for both clinical and anatomical issues. Jiang et al. provide us with data from a pediatric cohort of 6 patients aged 8.4 years, ±2.6 years, presenting with VA originating from the moderator band of the right ventricle. This paper enriches this special issue, representing a good example of how catheter ablation could also be effective in this insidious condition typical of young patients with VA originating in a difficult to access anatomical area. Access to catheter ablation is often limited by patients' hemodynamic instability, either due to the high number of arrhythmias or to the extent of left ventricular dysfunction. In this regard, the use of the PAINESD score has been proposed to identify patients more likely to be at risk of hemodynamic collapse during the procedure (2). According to this score, which is measured from 0 to 36 points, patients scoring more than 17 points showed a 24% risk of acute hemodynamic decompensation. Additionally, acute hemodynamic decompensation during the procedure was associated with a worse prognosis in the short-term. For these patients a mechanical circulatory support may be useful to maintain a good perfusion during catheter ablation and also to help with procedure-related complications.

This is the rationale behind two papers in this special issue. The first is a review by Tavazzi et al. that explains the physiology, workflow, and indications for the clinical use of mechanical left ventricular assist devices to support electrophysiological procedures. The second paper, by Delmas et al. describes a new type of percutaneous, and rather inexpensive, left ventricle assist device (IVAC2L by PulseCath^®^ Amsterdam; The Netherlands) that can be connected to any standard and widely available intra-aortic balloon pump drive-console. This new device can provide a pulsatile flow by aspirating from the left ventricle during systole and injecting into the aorta during diastole. Available data on this new technology comes mainly from animal studies on cardiogenic shock and its use to support electrophysiological procedures, although potentially very promising, still needs to be evaluated.

Despite newer technology, an increase in operators' experience and the potential use of circulatory support, ablation's success rate is still suboptimal and/or ablation may not be available in every center 24 h a day. Therefore, there is a need for alternative treatments to address VA, such as during an electrical storm. In the European guidelines already mentioned (1), for the first time neuromodulation is considered an additional weapon to halt electrical storms. This is an emerging field and represents the biggest breakthrough in modern emergency treatment of VA. Wu et al. explore the mechanisms underlying neuromodulation by demonstrating that by reducing the activity of sympathetic nerves that connect to the heart, the optical activation of the dorsal horn of the thoracic cord can prevent VA in an animal ischemia/reperfusion model. These results encourage a larger use of neuromodulation and, among the different ways to block sympathetic activation, percutaneous stellate ganglion block (3) is by far the easiest and the fastest for patients with electrical storm whose indications and clinical use should be encouraged (4).

After the acute phase, the goal is to prevent recurrences. With this in mind, the central role of sympathetic activation has been well-recognized in arrhythmias of genetic origin, such as in long QT syndrome, where sympathetic cardiac denervation has been proven to be highly effective in preventing clinical recurrences (5). The use of cardiac sympathetic denervation has been recently extended to the treatment of VA in patients with structural heart disease with very good results (6, 7). Additionally, stereotactic radiotherapy of VA is an emerging technique for the treatment of VA recurrences that has shown promising results (8, 9).

In this special issue, we are proud to present the work by Huang et al. describing the first case of recurrent VA effectively treated with a single 12-Gy dose. The future of this technique might be characterized by the use of less energy with a high safety profile and by the probable use of other energy sources as particle therapies (10). Little has been discovered in recent years about new antiarrhythmic drugs. One of the papers in this collection is a review by Wang X. et al. on the pharmacological effects of Xin Su Ning, a multicomponent medicine derived from traditional Chinese medicine. Cellular electrophysiological studies have shown how this medicine is both a sodium and potassium channel regulator similar to amiodarone. It plays a myocardial protective role during the ischemia/reperfusion phase, and has been shown to reduce the number of ectopic beats in patients with viral myocarditis. In a three-arm randomized clinical trial involving more than 800 patients, it performed like mexiletine by reducing the number of premature ectopic beats.

Many patients with a history of VA and/or a structural heart disease are implanted with a cardioverter defibrillator. In these patients, supraventricular arrhythmias may be a potential cause of inappropriate shocks, so their treatment is essential. Zhang et al. report on their 10-year experience of 34 supraventricular arrhythmias ablations in 101 patients with hypertrophic cardiomyopathy. They found no recurrences after the ablation of arrhythmias other than atrial fibrillation (AF) and observed a AF-free survival rate of 87.5 and 49.5% at 1 and 7 years, respectively, after AF ablation. To reduce recurrences, it is essential to validate the effectiveness of ablation. Wei et al. demonstrate how adenosine administration could help to confirm the ablation success of an accessory pathway.

In summary, treating patients with VA is one of the greatest challenges of modern cardiology. In recent times, more sophisticated tools are becoming available for cardiologists and electrophysiologists to use against VA. Deciding how and when to use these resources, such as pharmacological therapy, implantable cardioverter defibrillator, catheter ablation, left ventricular assist devices, neuromodulation, and potentially, radiotherapy, is a difficult task. We believe that this Research Topic, which we had the privilege of editing, will provide enlightening and original insights into this fascinating topic.

## Author contributions

SS and RR are responsible for conceptualizing, writing, and revising this paper. Both authors approved the submitted version.

## References

[1] Zeppenfeld K Tfelt-Hansen J de Riva M Winkel BG Behr ER Blom NA Charron P Corrado D. 2022 ESC guidelines for the management of patients with ventricular arrhythmias and the prevention of sudden cardiac death developed by the task force for the management of patients with death of the European society of cardiology (ESC) endorsed by the association for European paediatric and congenital cardiology (AEPC). Eur Heart J. (2022) 43:3997–4126. 10.1093/eurheartj/ehac262 36017572

[2] Santangeli P Muser D Zado ES Magnani S Khetpal S Hutchinson MD. Acute hemodynamic decompensation during catheter ablation of scar-related ventricular tachycardia: incidence, predictors, and impact on mortality. Circ Arrhythm Electrophysiol. (2015) 8:68–75. 10.1161/CIRCEP.114.002155 25491601

[3] Savastano S Dusi V Baldi E Rordorf R Sanzo A Camporotondo R. Anatomical-based percutaneous left stellate ganglion block in patients with drug-refractory electrical storm and structural heart disease: a single-centre case series. Europace. (2021) 23:581–6. 10.1093/europace/euaa319 33190159

[4] Savastano S Schwartz PJ. Blocking nerves and saving lives. Left stellate ganglion block for electrical storms. Heart Rhythm. (2022). 10.1016/j.hrthm.2022.11.025. [Epub ahead of print]. 36509320

[5] Dusi V Pugliese L De Ferrari GM Odero A Crotti L Dagradi F. Left cardiac sympathetic denervation for long QT syndrome: 50 years' experience provides guidance for management. JACC Clin Electrophysiol. (2022) 8:281–94. 10.1016/j.jacep.2021.09.002 35331422

[6] Savastano S Pugliese L Baldi E Dusi V Tavazzi G De Ferrari GM. Percutaneous continuous left stellate ganglion block as an effective bridge to bilateral cardiac sympathetic denervation. Europace. (2020) 22:606. 10.1093/europace/euaa007 32034906

[7] Dusi V Pugliese L Passarelli I Camporotondo R Driussi M Antonutti M. Bilateral cardiac sympathetic denervation in structural heart disease. Eur Heart J Suppl. (2019) 40:ehz746.0065. 10.1093/eurheartj/ehz746.0065

[8] Cuculich PS Schill MR Kashani R Mutic S Lang A Cooper D. Noninvasive cardiac radiation for ablation of ventricular tachycardia. N Engl J Med. (2017) 377:2325–36. 10.1056/NEJMoa1613773 29236642 PMC5764179

[9] Robinson CG Samson PP Moore KM Hugo GD Knutson N Mutic S. Phase I/II trial of electrophysiology-guided noninvasive cardiac radioablation for ventricular tachycardia. Circulation. (2019) 139:313–21. 10.1161/CIRCULATIONAHA.118.038261 30586734 PMC6331281

[10] Dusi V Vitolo V Frigerio L Totaro R Valentini A Barcellini A. First-in-man case of non-invasive proton radiotherapy for the treatment of refractory ventricular tachycardia in advanced heart failure. Eur J Heart Fail. (2021) 23:195–6. 10.1002/ejhf.2056 33179329

